# 806. *Candida* Gut Colonization in Critically Ill Patients

**DOI:** 10.1093/ofid/ofad500.851

**Published:** 2023-11-27

**Authors:** Max W Adelman, Truc T Tran, Andrea M Detranaltes, Kirsten B Rydell, Rachel Atterstrom, Marissa G Schettino, Abigail A Amaya, Husna Malikzad, Muhammad H Virk, Mary N Jones, Alex E Deyanov, Jose C Perez, David B Corry, Michael C Lorenz, Rodrigo de Paula Baptista, Cesar A Arias

**Affiliations:** Houston Methodist Hospital, Houston, Texas; Houston Methodist Hospital, Houston, Texas; Houston Methodist Hospital, Houston, Texas; Houston Methodist Hospital, Houston, Texas; Houston Methodist Hospital, Houston, Texas; Houston Methodist Hospital, Houston, Texas; Houston Methodist Hospital, Houston, Texas; Houston Methodist Hospital, Houston, Texas; Houston Methodist Hospital, Houston, Texas; Houston Methodist Hospital, Houston, Texas; Houston Methodist Hospital, Houston, Texas; UTHealth, Houston, Texas; Baylor College of Medicine, Houston, Texas; UTHealth, Houston, Texas; Houston Methodist Hospital, Houston, Texas; Houston Methodist and Weill Cornell Medical College, Houston, TX

## Abstract

**Background:**

*Candida* spp. colonization is a risk factor for candidemia in intensive care unit (ICU) patients. Recent data suggest that the epidemiology of *Candida* infections is shifting away from *C. albicans*, but prospective data on the epidemiology of *Candida* spp. gut colonization are limited.

**Methods:**

We conducted a prospective cohort study of adult patients admitted to an ICU at a tertiary care hospital. Patients had stool samples collected twice weekly for up to four weeks or until ICU discharge. A convenience sample of stool samples was plated on CHROMagar *Candida* Plus for 48 hours. Colonies of different morphologies were isolated on Sabouraud dextrose agar and identified with MALDI-ToF.

**Results:**

A total of 102 patients were included in the cohort. We performed screening for stool *Candida* colonization in 12 patients: 8 (67%) men, median age 65 (IQR 54.5-68.5). Three (25%) were in shock and eight (67%) required mechanical ventilation on ICU admission. In-hospital mortality was 25%. Seven patients (58%) were colonized with *Candida* at ≥1 time point (**Figure 1**). Of 30 total time points tested (median per patient=2, range 1-5), 15 (50%) were positive for *Candida* spp. Most time points (9/15, 60%) were positive for *C. glabrata*; 4/15 (27%) were positive for both *C. glabrata* and *C. albicans*. A minority (1/15; 7%) were positive for *C. albicans* and *C. parapsilosis* alone, respectively. None had *C. auris* colonization. Most colonization was persistent: Of 6 colonized patients with ≥1 time point tested, 5 (83%) were colonized at multiple timepoints. Eleven patients had data collected on anti-fungal administration during the study period, and five of these received an antifungal (**Figure 1**). Of these five, three (60%) had no *Candida* colonization. One lost *C. albicans* colonization but had persistent *C. glabrata* colonization despite azole therapy, and another developed *C. parapsilosis* colonization despite azole and amphotericin administration.

Figure 1.

**Figure:**
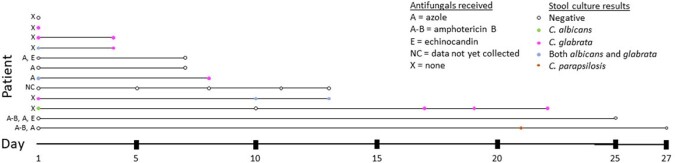

Results of serial stool samples tested for Candida colonization per individual patient.

**Conclusion:**

Most ICU patients in this study had *Candida* spp. gut colonization, commonly with intrinsically azole-resistant *C. glabrata*. This study is limited by small sample size, and further data are needed to determine the clinical impact of antifungal-resistant *Candida* in ICU patients at high risk of candidemia.

**Disclosures:**

**All Authors**: No reported disclosures

